# N-Cadherin Upregulation Promotes the Neurogenic Differentiation of Menstrual Blood-Derived Endometrial Stem Cells

**DOI:** 10.1155/2018/3250379

**Published:** 2018-03-05

**Authors:** Yanli Liu, Fen Yang, Shengying Liang, Qing Liu, Sulei Fu, Zhenyu Wang, Ciqing Yang, Juntang Lin

**Affiliations:** ^1^Stem Cell Research Center, College of Life Science and Technology, Xinxiang Medical University, Xinxiang 453003, China; ^2^Henan Key Laboratory of Medical Tissue Regeneration, Xinxiang 453003, China; ^3^College of Biomedical Engineering, Xinxiang Medical University, Xinxiang 453003, China; ^4^School of Biological and Chemical Engineering, Liaoning Institute of Science and Technology, Benxi 117004, China

## Abstract

Peripheral nerve injuries are typically caused by either trauma or medical disorders, and recently, stem cell-based therapies have provided a promising treatment approach. Menstrual blood-derived endometrial stem cells (MenSCs) are considered an ideal therapeutic option for peripheral nerve repair due to a noninvasive collection procedure and their high proliferation rate and immunological tolerance. Here, we successfully isolated MenSCs and examined their biological characteristics including their morphology, multipotency, and immunophenotype. Subsequent in vitro studies demonstrated that MenSCs express high levels of neurotrophic factors, such as NT3, NT4, BDNF, and NGF, and are capable of transdifferentiating into glial-like cells under conventional induction conditions. Moreover, upregulation of N-cadherin (N-cad) mRNA and protein expression was observed after neurogenic differentiation. In vivo studies clearly showed that N-cad knockdown via in utero electroporation perturbed the migration and maturation of mouse neural precursor cells (NPCs). Finally, a further transfection assay also confirmed that N-cad upregulation in MenSCs results in the expression of S100. Collectively, our results confirmed the paracrine effect of MenSCs on neuroprotection as well as their potential for transdifferentiation into glial-like cells and demonstrated that N-cad upregulation promotes the neurogenic differentiation of MenSCs, thereby providing support for transgenic MenSC-based therapy for peripheral nerve injury.

## 1. Introduction

Schwann cells (SCs) play a key role in the development, function, and regeneration of peripheral nerves. However, clinical application of SC transplantation is limited due to the intrinsic shortcomings of SCs, such as the invasive isolation requirements, their limited proliferation capacity in vitro, and their high immunogenicity [1, 2]. Recently, increasing evidence has suggested that adult stem cell (ASC) transplantation is an ideal alternative to SC transplantation and may be promising in the clinic [2–4]. Transplantation of undifferentiated ASCs has been demonstrated to be beneficial for peripheral nerve repair, most likely via the paracrine production of neurotrophic factors rather than the direct transdifferentiation into SCs. Therefore, to improve the benefit of ASC-based therapy for peripheral nerve repair, several studies have attempted to predifferentiate ASCs into Schwann-like cells in vitro prior to transplantation [5–7]. The main purpose of predifferentiation is to improve the survival of the predifferentiated ASCs residing at the injury site and promote their fusion with endogenous SCs while simultaneously reducing the possibility of differentiation of the transplanted cells into undesired cell types.

As a class of newly defined ASCs, menstrual blood-derived endometrial stem cells (MenSCs) show promise for clinical application. MenSCs are mesenchymal-like stem cells that can be harvested from human menstrual blood shed from the endometrium every month and have a high proliferation and differentiation capability under specific differentiation conditions. The convenience of obtaining MenSCs without invasive surgical intervention or hospitalization and the absence of any ethical issues associated with their isolation provide reasonable advantages for the clinical application of MenSCs [8–10]. In addition to being positive for classical mesenchymal stem cell markers (CD29, CD44, CD73, CD90, and CD105) and negative for hematopoietic cell surface markers (CD34, CD45, and CD133), MenSCs also express several pluripotency markers, including OCT-4, SOX2, and SSEA-4 [11]. Differentiation of MenSCs into adipocytes, chondrocytes, osteocytes, hepatocytes, cardiomyocytes, and pancreatic cells has been demonstrated previously. The promising therapeutic potential of MenSCs has been demonstrated in central nerve repair (using experimental mouse models of stroke and Parkinson's disease), and the safety of MenSC administration has been evaluated [10, 12, 13].

N-Cadherin (N-cad) is primarily expressed in neural tissues and plays a critical role in the development of the nervous system. N-cad is responsible for regulating maintenance, proliferation, and differentiation of neural precursor cells (NPCs) during development [14–16]. Based on earlier studies, predifferentiation of MenSCs into Schwann-like cells may be more beneficial for peripheral nerve repair; however, the toxicity of chemical factors and the high cost of biological factors for the predifferentiation process vastly limit its clinical application. Therefore, our study aims at first confirming the paracrine effect of MenSCs on neuroprotection and their potential for transdifferentiation into glial-like cells and, subsequently, at examining alterations in the level of N-cad during predifferentiation of MenSCs to explore an alternative source of genetically engineered MenSCs for treating peripheral nerve injury.

## 2. Materials and Methods

### 2.1. Plasmids, Cells, and Animals

The plasmids (pCAG-MCS-EGFP and human pCAG-N-cad-EGFP) were constructed in our laboratory. The MenSCs used in this study were harvested with informed consent from female donors, and this study was approved by the Ethics Committee of Xinxiang Medical University. Eight-week-old BALB/c mice (20–25 g) were purchased from Vital River Laboratories (Beijing, China) and bred and housed in a pathogen-free environment with a 12-hour light-dark cycle in the animal care facility. All experimental protocols were approved by the Animal Research Committee of Xinxiang Medical University according to the Chinese Council on Animal Care guidelines.

### 2.2. Isolation and Culture of MenSCs

Samples of menstrual blood were collected from healthy female donors (*n* = 5, 30 ± 5 years of age) with menstrual cups during the first three days of menses and quickly mixed with equal volumes of PBS containing 0.25 mg/ml amphotericin B, 100 U/ml penicillin, 100 mg/ml streptomycin, and 2 mM EDTA. Once the microbial contaminants were eliminated, MenSCs were isolated within 72 h using the standard Ficoll method as previously described [8], suspended in growth medium [high-glucose DMEM (HyClone, USA) supplemented with 10% FBS (Gibco, USA), 100 U/ml penicillin, and 100 mg/ml streptomycin], and seeded in T25 flasks at 37°C, 5% CO_2_. After 2 days of incubation, the nonadherent cells were washed away, and the growth medium was replaced every 3 days. When the cells reached 80–90% confluence (passage 0, P0), the cells were detached with trypsin and subcultured to new flasks at a ratio of 1 : 3.

### 2.3. Identification of MenSCs

The classical characteristics of adult stem cells, including in vitro multilineage differentiation potential and the typical immunophenotype, were routinely examined in P3 MenSCs, and each examination was repeated separately on MenSCs isolated from 3 donors (*n* = 3). In brief, the morphology of MenSCs (P0, P3, and P9) was determined by imaging; the expression of surface markers, such as CD29, CD44, CD73, CD90, CD105, HLA-ABC, HLA-DR, CD34, and CD45, was tested using FACS. Adipogenic, osteogenic, and chondrogenic differentiation was induced and identified as follows: adipogenic differentiation medium (growth medium + 1 *μ*mol/l dexamethasone + 10 *μ*g/ml recombinant human insulin + 200 *μ*M indomethacin + 0.5 mM IBMX), for 14 days; osteogenic differentiation medium (growth medium + 0.1 *μ*mol/l dexamethasone + 0.05 mmol/l ascorbic acid + 10 mM *β*-glycerophosphate), for 21 days; and chondrogenic differentiation medium (growth medium + 0.1 *μ*mol/l dexamethasone + 0.2 mmol/l ascorbic acid + 1% insulin-transferrin-selenic acid + 10 ng/ml TGF-*β*3), for 21 days. At the end of the induction period, the cells were washed and fixed. Adipogenic differentiation was confirmed by Oil red O staining, osteogenic differentiation was confirmed by Alizarin red staining, and chondrogenic differentiation was confirmed by Alcian blue staining.

### 2.4. In Vitro Neurogenic Differentiation Assay

The P3 MenSCs (*n* = 3, the MenSCs were isolated from 3 donors) were suspended in growth medium and seeded at a density of 2 × 10^4^ cells/well in a 6-well plate until confluence. Then, the growth medium was changed to glial differentiation medium (growth medium + 1 mM *β*-mercaptoethanol, for 1 day; growth medium + 35 ng/ml all-trans-retinoic acid, for 3 days; growth medium + 5 ng/ml platelet-derived growth factor + 10 ng/ml basic fibroblast growth factor + 14 *μ*M forskolin + 126 ng/ml glial growth factor-2, for 14 days). The control cells were cultured in growth medium, and the medium was replaced every 3 days.

### 2.5. RT-PCR

Total RNA was isolated from cells with or without neurogenic differentiation (2 × 10^6^), and cDNA was prepared from 2 *μ*g of total RNA using PrimeScript RT Master Kit according to the manufacturer's instructions. The primers used to amplify the target genes were synthesized as listed in Table 1; the housekeeping gene (GAPDH) was used as an internal control. The PCR products were amplified using 2× Taq Master Mix (Cwbiotech, China) and analyzed by electrophoresis on 2% agarose gels and with ethidium bromide staining. The final data were normalized against the intensity (gray value) of the GAPDH signal and are expressed as mRNA expression relative to that of GAPDH.

### 2.6. Immunofluorescence

The cells were fixed in 4% PFA for 20 min and permeabilized with 0.05% Triton X-100 for 10 min; nonspecific binding was blocked with 5% goat serum for 30 min. Anti-S100, anti-GFAP, and anti-N-cad (Abcam, USA) antibodies were separately added, and the cells were incubated at 4°C overnight. Alexa Fluor 488- and Cy3-conjugated goat anti-mouse or anti-rabbit secondary antibodies (Life Technologies, USA) were incubated with the cells at 37°C for 1 h. Cell nuclei were stained with DAPI. Finally, the cells were observed and imaged under an inverted fluorescence microscope (Leica, Germany). After neurogenic differentiation, the percentages of GFAP-/S100-/N-cad-positive cells in MenSCs were quantified based on the 10 images collected randomly.

### 2.7. In Utero Electroporation

The protocol for in utero electroporation (IUE) was followed as previously described [24]. The pGPU6-GFP-neo-shRNA-N-cad (plasmid-based shRNA) was ordered from Kangwei (Abgent, Jiangsu, China) based on the specific shRNA sequence for mouse N-cad (5′-GCCTATGAAGGAACCACATGA-3′), and pGPU6-GFP-neo-shRNA-Control served as the negative control. To strengthen the visible effect, an 8 : 1 ratio of the plasmid-based shRNA and pCAG-MCS-EGFP plasmid was used due to the weak intensity of GFP in mouse embryos induced by plasmid-based shRNA. Solutions (1 *μ*l each) containing pCAG-MCS-EGFP plasmid (0.5 *μ*g) and pGPU6-GFP-neo-shRNA-N-cad or pGPU6-GFP-neo-shRNA-Control (4 *μ*g) were injected through the uterine wall into the lateral ventricle of the embryos at E15 (embryonic day 15). After injection, paddle-type electrodes were placed on either side of the head of the embryo, and five 60 ms square pulses with 600 ms intervals at 35 V were applied with an electroporator (NAPA gene, CUY21, Japan). The electroporated embryos were further incubated for five days, and GFP-positive brains (*n* = 3) were collected for investigation at E20 using a stereo fluorescence microscope (Leica M205FA, Germany). Brain cryosections were prepared according to a previously described protocol, and sections were imaged under a fluorescence microscope (Nikon ECLIPSE 80i, Japan) equipped with digital camera (Leica DFC300FX, Germany).

### 2.8. Transfection

MenSCs (*n* = 3, the MenSCs were isolated from 3 donors) were transfected with Lipofectamine™ 3000 reagent (Invitrogen Co. Ltd., USA) following the manufacturer's protocol. In brief, MenSCs were seeded at a density of 1 × 10^5^ cells/well into a 24-well plate 24 h before transfection. Subsequently, a final volume of 50 *μ*l of serum free DMEM containing 500 ng of plasmid (pCAG-MCS-EGFP or human pCAG-N-cad-EGFP), 1 *μ*l P3000™ reagent, and 1.5 *μ*l Lipofectamine 3000 reagent was added to each well. The medium was replaced with fresh medium (high-glucose DMEM supplemented with 2% FBS) every 3 days, and GFP fluorescence was detected with an inverted fluorescence microscope. After being cultured for 14 days, the cells were harvested and analyzed via immunofluorescence.

### 2.9. Statistical Analysis

The data are presented as the mean ± SD; all experiments were repeated at least in duplicate on MenSCs isolated from 3 donors; and the figures presented in the manuscript show representative images. Student's *t*-test was used to determine statistical significance, and *p* < 0.05 was considered statistically significant.

## 3. Results

### 3.1. Isolation and Identification of MenSCs

**Figure 1:**
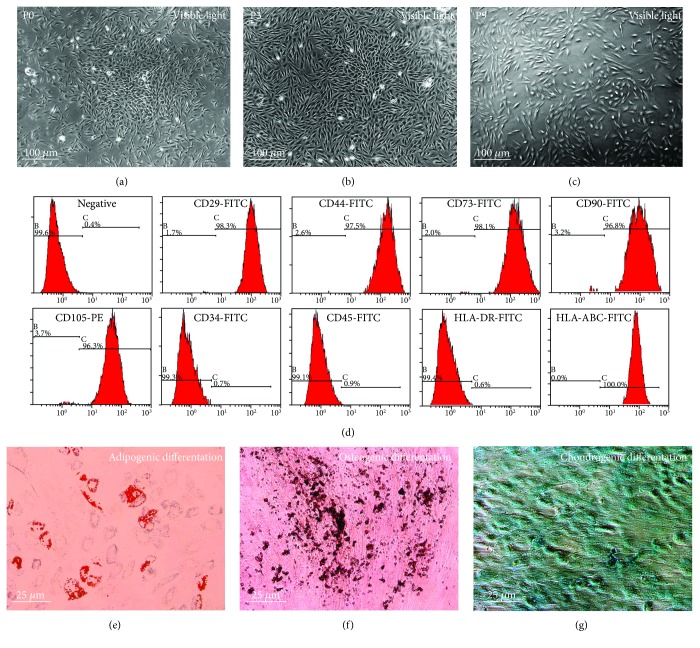
Isolation and identification of MenSCs. Phase contrast images showing the morphology of primary MenSCs at different passages: (a) P0, (b) P3, and (c) P9. (d) The phenotype of MenSCs. To determine the immunophenotype of the cells, P3 MenSCs were stained with the indicated conjugated antibodies and analyzed via FACS. The P3 MenSCs were positive for classical ASC markers (CD29, CD44, CD73, CD90, and CD105) and HLA-ABC and negative for hematopoietic stem cell markers (CD34 and CD45) and HLA-DR. (e) Adipogenic, (f) osteogenic, and (g) chondrogenic differentiation was conventionally induced, and the results were assessed with positive Oil red O, Alizarin red, and Alcian blue staining, respectively.

After isolation using the standard Ficoll method, a colony-like morphology was clearly observed in the primary cultures of MenSCs (Figure 1(a)), and the subcultured MenSCs demonstrated growth characteristics typical of ASCs (spindle fibroblast-like morphology with a radial or helical growth pattern). Moreover, the identified phenotype was stable throughout the subculture period from P0 to P9 (Figures 1(a)–1(c)). Subsequently, flow cytometric analysis of P3 MenSCs demonstrated that the cultured cells were positive for CD29, CD44, CD73, CD90, CD105, and HLA-ABC but negative for CD34, CD45, and HLA-DR (Figure 1(d)). Finally, multilineage differentiation assays also confirmed that the MenSCs could undergo adipogenic (Figure 1(e)), osteogenic (Figure 1(f)), and chondrogenic (Figure 1(g)) differentiation after being treated with specific induction media.

### 3.2. Paracrine Effect of MenSCs on Neuroprotection

**Figure 2:**
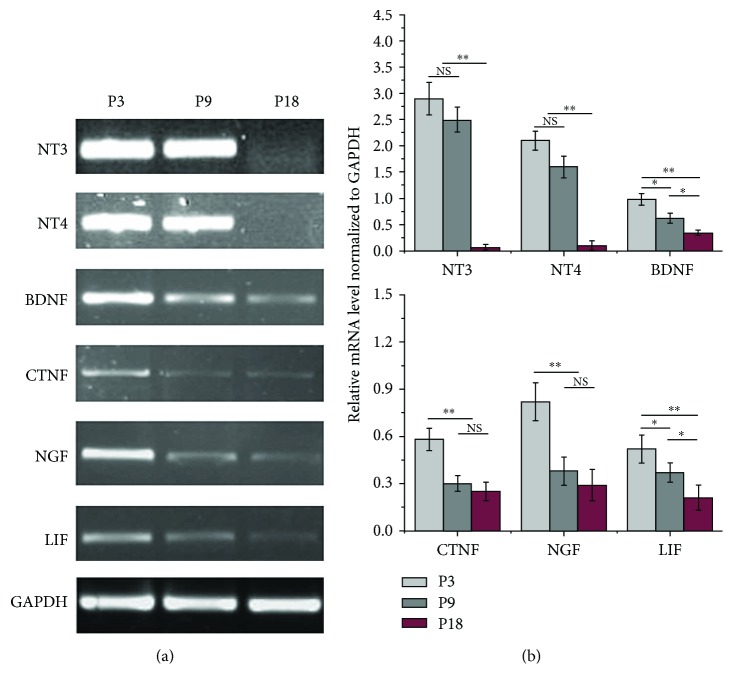
Expression of neurotrophic genes at different passages (P3, P9, and P18) of MenSCs cultured in vitro. (a) RT-PCR analysis of neurotrophic factors expressed at different passages in MenSCs. (b) Quantitative analysis of neurotrophic factors expressed at different passages in MenSCs. The data were normalized against the intensity (gray value) of GAPDH expression and are expressed as the expression level of mRNA relative to that of GAPDH. ^∗^*p* < 0.05; ^∗∗^*p* < 0.01.

RT-PCR analysis of neurotrophic factors (Figure 2) revealed a high level of NT3, NT4, BDNF, and NGF expressions compared with the level of CTNF and LIF in the P3 MenSCs cultured in vitro. However, with an increase in culture time, the expression of the neurotrophic factors mentioned above was significantly decreased in the MenSCs, especially at P18, which suggested that the MenSCs passaged earlier were optimal for peripheral nerve repair.

### 3.3. Differentiation of MenSCs into Glial-like Cells

**Figure 3:**
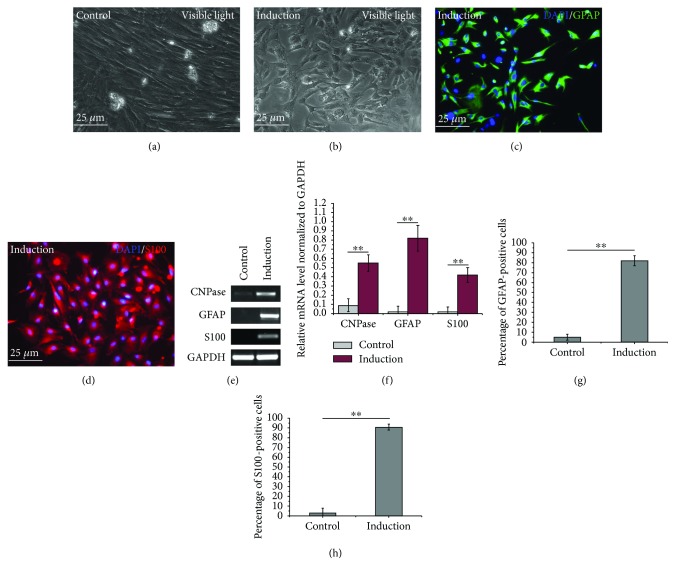
Differentiation of MenSCs into glial-like cells. (a, b) Morphology of P3 MenSCs with or without neurogenic induction. (c, d) Neurogenic differentiation of P3 MenSCs was induced, and the results were assessed by examining the positive expression of glial cell markers (GFAP and S100). (e, f) RT-PCR analysis of CNPase, GFAP, and S100 in MenSCs with or without neurogenic induction. (g, h) The percentage of GFAP- or S100-positive cells after neurogenic differentiation. ^∗∗^*p* < 0.01.

In accordance with previous reports, the neurogenic differentiation potential of MenSCs was confirmed in this study. After induction of neural differentiation, the morphology of the MenSCs was transformed from the original spindle fibroblast-like morphology into a glial-like morphology (Figures 3(a) and 3(b)). Subsequent immunofluorescence analysis demonstrated that the differentiated MenSCs were positive for GFAP and S100 (>80%, Figures 3(c), 3(d), 3(g), and 3(h)), which are well-recognized markers of glial cells, and RT-PCR results showed increased mRNA levels of CNPase, S100, and GFAP (Figures 3(e) and 3(f)).

### 3.4. Neurogenic Differentiation of MenSCs Promotes N-cad Expression

**Figure 4:**
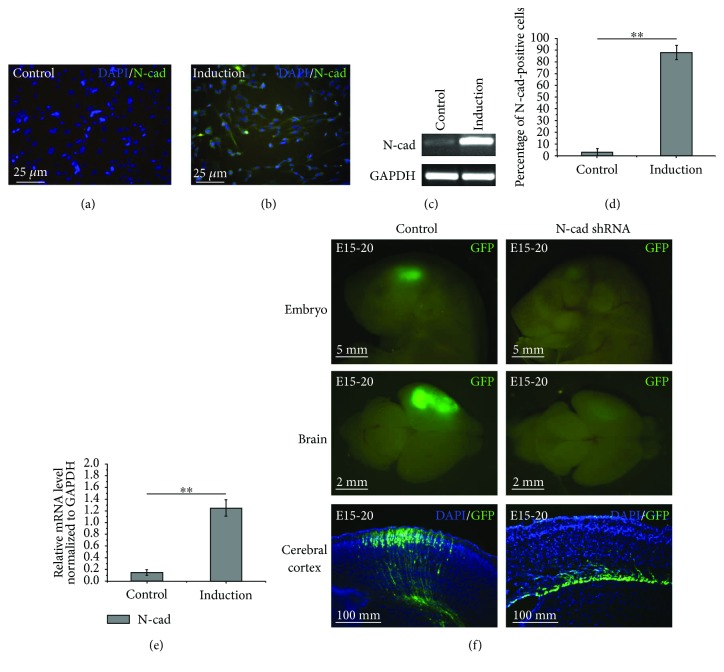
(a-e) Neurogenic differentiation of MenSCs promotes N-cad expression. Immunofluorescence staining (a, b) and RT-PCR (c) were used to determine the expression of N-cad in MenSCs with or without neurogenic induction. The percentage of N-cad-positive cells (d) and the relative mRNA expression levels of N-cad (e) in MenSCs after neurogenic differentiation. (f) Inhibition of N-cad expression affects the migration of NPCs in vivo. The GFP-positive embryos and brains were collected and imaged under a stereo fluorescence microscope at E20, and the subsequently prepared cryosections clearly showed that the decrease in GFP intensity was caused by abnormal retention of GFP-positive NPCs at the ventricular zone. ^∗∗^*p* < 0.01.

The immunofluorescence and RT-PCR results demonstrated the expression of N-cad in the MenSCs after neurogenic differentiation (Figures 4(a)–4(e)). Compared to the undifferentiated MenSCs, the expression of N-cad was significantly increased in the differentiated MenSCs at both the mRNA and protein levels, which suggests that N-cad plays a role during neurogenic differentiation of MenSCs.

### 3.5. Inhibition of N-cad Expression Affects the Migration of Neural Progenitor Cells

Equal amounts of pGPU6-GFP-neo-shRNA-N-cad and pGPU6-GFP-neo-shRNA-Control were transfected into the center of the cerebral hemisphere to target the lateral ventricle at E15 by IUE. Then, the GFP-positive embryos and brains were collected and imaged under a stereo fluorescence microscope at E20. As shown in Figure 4(f), compared with the negative control group, the intensity of GFP in the pGPU6-GFP-neo-shRNA-N-cad-transfected embryos and brains was visibly decreased, and the subsequently prepared cryosections clearly showed that the decrease in GFP was caused by abnormal retention of GFP-positive NPCs at the ventricular zone (VZ), which suggests that inhibition of N-cad expression significantly influences NPC migration and maturation.

### 3.6. Upregulation of N-cad Promotes Neurogenic Differentiation of MenSCs

**Figure 5:**
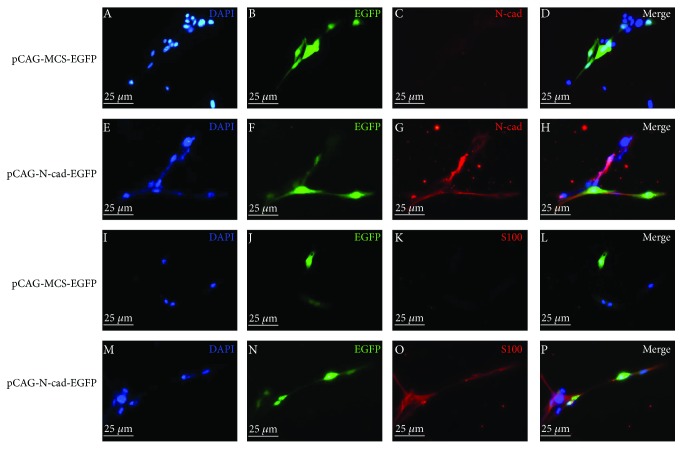
The promotive effect of N-cad upregulation on neurogenic differentiation of MenSCs. MenSCs were transfected with different plasmids, and the medium was replaced with fresh medium every 3 days. After cells were cultured for 14 days, immunofluorescence was performed. (a-d) and (i-l) EGFP-positive MenSCs in the pCAG-MCS-EGFP-treated group were negative for N-cad and S100 expressions. (e-h) and (m-p) EGFP-positive MenSCs in the human pCAG-N-cad-EGFP-treated group were positive for N-cad and S100 expressions.

To determine whether upregulation of N-cad could promote neurogenic differentiation of ASCs, MenSCs were transiently transfected with the N-cad overexpression vector (human pCAG-N-cad-EGFP), and the immunofluorescence results are shown in Figure 5. After transfection, EGFP-positive MenSCs in the human pCAG-N-cad-EGFP-treated group were positive for N-cad (Figure 5(g)) and S100 (Figure 5(o)) expressions, but the cells in the pCAG-MCS-EGFP-treated group were negative for N-cad (Figure 5(c)) and S100 (Figure 5(k)) expressions.

## 4. Discussion

The high incidence of peripheral nerve injury represents a major economic and physiological burden for patients due to poor functional recovery [25]. Fortunately, ASC-based therapies are being established via experimental and clinical studies, and considerable progress has been made in the past decades, which offers hope for diseases that lack effective treatments [26, 27]. MenSCs, a newly identified class of ASCs, have potential for peripheral nerve repair because of essential advantages such as abundant sources, noninvasive isolation procedures, lack of ethical controversy, and high neurogenic transdifferentiation potency [8–13]. Our in vitro studies not only analyzed the paracrine effect of MenSCs on neuroprotection (Figure 2) but also confirmed that MenSCs could be predifferentiated into Schwann-like cells with conventional induction protocols (chemicals combined with biological factors), which demonstrated the neurogenic differentiation capacity of MenSCs and suggested their potential to transdifferentiate into Schwann-like cells in vivo. The above results showed the therapeutic potential of MenSCs transplantation for peripheral nerve regeneration.

Furthermore, several studies have reported that compared with transplantation of naïve ASCs, transplantation of predifferentiated ASCs is more promising and could improve the survival of the transplanted cells at the injury site and promote their fusion with endogenous SCs, while simultaneously reducing the possibility of differentiation of the transplanted cells into undesired cell types [5–7]. However, to maintain the differentiation of ASCs into Schwann-like cells in vitro, chemical inducers combined with a rather expensive mixture of biological factors are often used, and the induction procedure is extremely complex and time-consuming. Although, coculture with SCs is a simple way to differentiate ASCs into Schwann-like cells, culturing SCs in vitro is difficult because they are often contaminated with fibroblasts present in the axolemma [3, 28–30]. Consequently, predifferentiation of ASCs to Schwann-like cells through a transgenic technique may be a promising alternative approach.

N-cad-based adherens junctions (AJs) are known to be involved in various neural development processes, such as neurulation, migration of neurons, elongation and guidance of axons, and synaptogenesis, due to the contribution of AJs to cell-cell adhesion between NPCs and neurons [14]. As research in this field continues, apart from the contribution to the integrity of AJs and apicobasal polarity of NPCs, N-cad is believed to be responsible for regulating maintenance, proliferation, and differentiation of NPCs during nervous system development [15, 16]. A substantial amount of data has suggested that the onset of neurogenic differentiation is characterized by downregulation of N-cad in various NPCs. It has been reported that the N-cad downregulation promotes detachment of apical processes from the VZ of the spinal cord of chick embryo and an abnormal persistence of N-cad expression inhibits the withdrawal of the apical process and cell cycle exit in prospective neurons [31, 32]. Furthermore, experiments using zebra fish models have shown that downregulation of N-cad is a trigger for differentiation of NPCs during rostral migration [33]. However, in contrast to neurogenic differentiation of NPCs, immediate expression of N-cad in induced pluripotent stem cells (iPSCs) substantially enhances the efficiency of neurogenic differentiation, which suggests that early activation of N-cad determines its potent neurogenic differentiation-promoting effect; subsequent knockdown experiments also confirmed that inhibition of N-cad expression by shRNA can block neurogenic differentiation [34].

Therefore, based on the results of previous studies and our preliminary findings, we examined the changes in N-cad expression after neurogenic differentiation of MenSCs, and the results showed upregulation of N-cad at both the mRNA and protein levels, suggesting the potential role of N-cad during neurogenic differentiation. In addition, our in vivo study confirmed that knockdown of N-cad by IUE perturbed the migration and maturation of mouse NPCs, which is in accordance with previous reports indicating that N-cad is needed to orient the migration of multipolar cells toward the cortical plate and that the potential mechanism is regulated by Jossin et al [35]. A further transfection assay confirmed that upregulation of N-cad in MenSCs indeed results in the expression of the glial cell marker S100, which indicated the potent neurogenic differentiation-promoting effect of upregulating N-cad in ASCs. Based on published data, the potent neurogenic differentiation-promoting effect of N-cad is likely mediated by *β*-catenin and Notch signaling pathways, which have been summarized in detail in several excellent reviews and papers [15, 16, 36]. Additionally, N-cad regulates the distribution and degradation of *β*-catenin, and GSK3*β*/Akt signal transduction may play critical roles during this regulation [15, 37]. Therefore, we postulate that the expression level of N-cad is likely to play an important role in neurogenic differentiation of MenSCs by regulating the structure of the cytoskeleton, which will indirectly influence the polarity and biomechanics of cells, and consequently promote neurogenic differentiation of MenSCs.

In conclusion, our results confirmed the paracrine effect of MenSCs on neuroprotection and their potential of transdifferentiation into glial-like cells. Moreover, we demonstrated that upregulation of N-cad could promote neurogenic differentiation of MenSCs. Future studies will be performed to validate the potential mechanisms and effect of transgenic MenSCs-based therapy in promoting peripheral nerve injury repair.

## Figures and Tables

**Table 1:** Primer sequences.

Gene	Oligonucleotide sequence (5′-3′)		Size (bp)	Reference
NT3	Sense	TACGCGGAGCATAAGAGTCAC	333	[17]
	Antisense	GGCACACACACAGGACGTGTC		
NT4	Sense	CTTTCGGGAGTCAGCAGGTGC	399	[17]
	Antisense	CAGGCAGTGTCAATTCGAATCC		
BDNF	Sense	TTCCACCAGGTGAGAAGAGT	474	[18]
	Antisense	ACTAATACTGTCACACACGC		
CNTF	Sense	TGGCTAGCAAGGAAGATTCGT	519	[19]
	Antisense	AATATAATGGCTCCCACGTGC		
NGF	Sense	CACACTGAGGTGCATAGCGT	389	[18]
	Antisense	TGATGACCGCTTGCTCCTGT		
LIF	Sense	ATGTCACAACAACCTCATGAA	466	[19]
	Antisense	GATCTGCTTATACTTCCCCAG		
	Antisense	AATGGTGATCCGGTTCTCCTC		
CNPase	Sense	AAGGACTTCCTGCCGCTCTA	466	[20]
	Antisense	TGTCCACATCACTCGGCCAC		
S100	Sense	ATGTCTGAGCTGGAGAAGG	338	[21]
	Antisense	CTGTCTGCTTTCTTGCATG		
GFAP	Sense	GTGGTACCGCTCCAAGTTTGCAG	376	[17]
	Antisense	AATGGTGATCCGGTTCTCCTC		
N-cad	Sense	TGTTTGACTATGAAGGCAGTGG	151	[22]
	Antisense	TCAGTCATCACCTCCACCAT		
GAPDH	Sense	GAAGGTGAAGGTCGGAGT	226	[23]
	Antisense	GAAGATGGTGATGGGATTTC		
